# Exploring the variability of sarcopenia prevalence in a research population using different disease definitions

**DOI:** 10.1007/s40520-023-02496-7

**Published:** 2023-07-19

**Authors:** Jessica Cegielski, Joseph J. Bass, Ruth Willott, Adam L. Gordon, Daniel J. Wilkinson, Ken Smith, Philip J. Atherton, Bethan E. Phillips

**Affiliations:** 1 Centre of Metabolism, Ageing and Physiology (COMAP), MRC-Versus Arthritis Centre for Musculoskeletal Ageing Research, National Institute for Health Research (NIHR) Nottingham Biomedical Research Centre, University of Nottingham, Nottingham, UK; 2 Department of Medicine for the Elderly, Royal Derby Hospital, Derby, UK; 3 Academic Unit of Injury, Recovery and Inflammation Sciences (IRIS), Centre of Metabolism, Ageing and Physiology (COMAP), School of Medicine, Faculty of Medicine and Health Sciences, University of Nottingham, Royal Derby Hospital Centre (Room 3011), Derby, DE22 3DT UK

**Keywords:** Ageing, Sarcopenia, Muscle, Health-span, Definitions

## Abstract

**Background:**

Sarcopenia is the progressive loss of muscle mass and function with age. A number of different sarcopenia definitions have been proposed and utilised in research. This study aimed to investigate how the prevalence of sarcopenia in a research cohort of older adults is influenced by the use of independent aspects of these different definitions.

**Methods:**

Data from 255 research participants were compiled. Defining criteria by the European Working Group on Sarcopenia in Older People, the International Working Group on Sarcopenia (IWGS), and the Foundation for the National Institutes of Health were applied.

**Results:**

Prevalence of sarcopenia using muscle mass ranged from 4 to 22%. Gait speed and handgrip strength criteria identified 4–34% and 4–16% of participants as sarcopenic, respectively.

**Conclusion:**

Prevalence of sarcopenia differs substantially depending on the criteria used. Work is required to address the impact of this for sarcopenia research to be usefully translated to inform on clinical practice.

## Introduction

Sarcopenia describes loss of muscle mass and muscle strength or function with advancing age [1]. In 2016, sarcopenia was classified by the World Health Organisation as a disease [2] and afforded an ICD-10 code as a “disorder of muscle”. Sarcopenia is associated with an increased risk of frailty, falls, and physical disability [3]. As such, identifying individuals who are at-risk of sarcopenia, or who are sarcopenic, has been proposed as the basis of selecting people for interventions to mitigate sarcopenia [4].

**Fig. 1:**
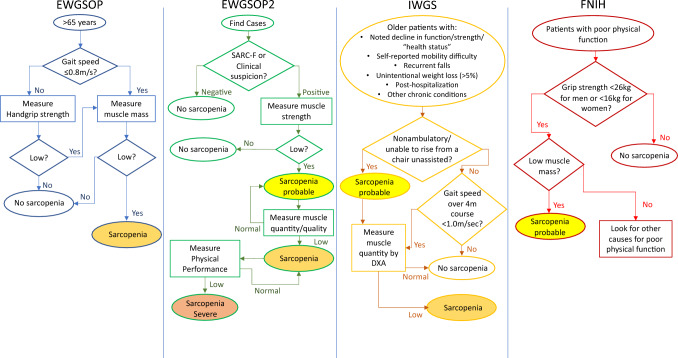
Sarcopenia diagnostic pathways provided by the European Working Group on Sarcopenia in Older People (EWGSOP), including their updated version (EWGSOP2), the International Working Group on Sarcopenia (IWGS) and the Foundation of National Institutes of Health (FNIH)

There are numerous definitions to assess and define sarcopenia. The most commonly used criteria are those proposed by the European Working Group on Sarcopenia in Older People (EWGSOP) [5], the International Working Group on Sarcopenia (IWGS) [6], and the Foundation of National Institutes of Health (FNIH) [7]; with a revised version of the EWGSOP definition published in 2018 (Fig. 1) [8]. These definitions each differ with respect to the cut-off values used for muscle mass and/or function. Although a number of studies have investigated the differences between these definitions in their entirety [9–12], the impact of different criteria within and between definitions in the context of an older research population has not been explored.

## Methods

Data from 255 male (155) and female (100) research participants aged 18–35 or over 65 years were used for this study (Table 1). All participants were independent, community-dwelling, and free from overt disease. All participants gave written, informed consent to participate in a specified research study (all of which were approved by the University of Nottingham Faculty of Medicine and Health Sciences Research Ethics Committee and complied with the Declaration of Helsinki) and for their data to be used in future research (i.e. such as that reported herein).

**Table 1:** Subject characteristics

	Young (18–35 years)				Older (65 years +)			
	Male		Female		Male		Female	
	Mean	*n*	Mean	*n*	Mean	*n*	Mean	*n*
Age (years)	24.0 ± 3.5	57	25.0 ± 3.6	26	71.5 ± 4,2	119	68.2 ± 2.6^^,^*	74
Height (m)	1.8 ± 0.1	57	1.6 ± 0.3^**^**^	26	1.74 ± 0.1*	119	1.6 ± 0.1^**^**^	74
Weight (kg)	77.5 ± 11.4	57	68.8 ± 12.6^**^**^	26	80.4 ± 10.4	119	67.1 ± 10.7*	74
BMI (kg/m^2^)	23.8 ± 3.1	57	24.9 ± 3.8	26	26.5 ± 2.9*	119	26.0 ± 3.7	74
FFM (kg)	56.2 ± 6.6	57	41.9 ± 5.6^**^**^	26	53.4 ± 5.3*	119	38.4 ± 4.5^^,^*	74
AFFM (kg)	27.3 ± 3.5	47	18.8 ± 3.6^^^	23	24.1 ± 3.7*	108	16.5 ± 2.1*	76
ASMI (kg/m^2^)	8.42 ± 0.92	47	6.87 ± 0.95^**^**^	23	7.94 ± 1.08	108	6.36 ± 0.67*	76
BMD (g/cm^2^)	1.31 ± 0.1	57	1.23 ± 0.1^^^	26	1.28 ± 0.13	119	1.07 ± 0.12^^,^*	68
BMC (g)	3154 ± 397.8	57	2510 ± 347.9^^^	26	3058 ± 411.4	115	2225 ± 398.2^^,^*	65
% Fat mass	22.6 ± 6.9	57	34.3 ± 6.9^^^	26	29 ± 5.8*	119	39.0 ± 5.8^^,^*	74

Values displayed as mean ± SD. Statistically significant between sex differences represented as ^; between age differences represented as *

*BMI* body mass index, *FFM* fat-free mass, *AFFM* appendicular FFM, *ASMI* appendicular skeletal mass index, *BMD* bone mineral density, *BMC* bone mineral content

All participants underwent a whole-body dual-energy X-ray absorptiometry (DXA) scan (Lunar Prodigy, GE Medical Systems, USA) for the determination of lean mass. Muscle function was assessed in older participants only, via handgrip strength (HGS, Takei, T.K.K. 5401 GRIP-D) and the Short Physical Performance Battery (SPPB) [13].

Statistical analyses were performed using Prism (GraphPad Software, San Diego, USA). Tukey’s and Dunn’s multiple comparison tests, for parametric and nonparametric variables, respectively, were used to identify significance differences between groups. Significance was set at *p* < 0.05.

## Results

Large differences in the proportion of the cohort identified as sarcopenic were found when using the different criteria for muscle mass alone (older females (OF): 8–22%, older males (OM): 4–16%, young (YF): 0–17%, and young males (YM): 0–11%) (Table 2). Similarly, using different accepted criteria for muscle mass from the *same* definition markedly altered prevalence (e.g. FNIH criteria for ALM: 8% vs. 22% for ALM adjusted for BMI) (Table 2).

**Table 2:** Prevalence of individuals meeting lean mass criteria in differing sarcopenia definitions

	Cut-off values	Young (18–35 years)		Older (> 65 years)	
		% Male sarcopenic	% Female sarcopenic	% Male sarcopenic	% Female sarcopenic
FNIH					
ALM adjusted for BMI	< 0.789 males	0	0	13	8
	< 0.512 females				
ALM	< 19.75 kg males	0	17	4	22
	< 15.02 kg females				
IWGS					
ALM/ht^2^ From: ^*a*^	< 7.26 kg/m^2^ males	11	17	16	12
	< 5.67 kg/m^2^ females				
EWG SOP2					
ALM/ht^2^	< 7.0 kg/m^2^ males	4	9	10	10
	< 5.5 kg/m^2^ females				
ALM	< 20 kg males	0	17	6	22
	< 15 kg females				
EWG SOP					
ALM/ht^2^ From: ^b^	< 7.26 kg/m^2^ males	11	9	16	9
	< 5.45 kg/m^2^ females				
ALM/ht^2^ From: ^c^	< 7.25 kg/m^2^ males	9	17	16	12
	< 5.67 kg/m^2^ females				
ALM/ht^2^ From: ^d^	< 7.32 kg/m^2^ males	9	17	16	12
	< 5.67 kg/m^2^ females				
	*n*	47	23	108	77
	SD	5.04	6.33	4.91	5.53

Definitions from the Foundation of National Institutes of Health (FNIH), the International Working Group on Sarcopenia (IWGS), and the European Working Group on Sarcopenia in Older People (EWGSOP), including their updated version (EWGSOP2)

*ALM* appendicular lean mass, *BMI* body mass index

^a^From Health Ageing and Body Composition (ABC) baseline cohort by Newman et al., 2003; ^b^From Rosetta study by Baumgartner et al., 1998; ^c^Based on sex-specific lowest 20% by Delmonico et al., 2007; ^d^From Health ABC sex-specific lowest 20% by Newman et al., 2003

Considering muscle function, the revised criteria by the EWGSOP2 for HGS reduced the number of OM and OF identified as sarcopenic by 75% and 50%, respectively (Table 2), with the revised criteria identical to that by the FNIH. Applying the identical EWGSOP and FNIH gait speed criteria identified 2 OM and 1 OF as sarcopenic, compared to the IWGS criterion which identified 12 OM and 6 OF. For both males and females, and using each definition, more participants were identified as sarcopenic using gait speed compared to HGS (Table 2).

## Discussion

The aim of this study was to determine the impact of using the individual criteria from the four most commonly used definitions of sarcopenia in the same cohort of research participants in relation to sarcopenia prevalence. We found that not only did prevalence of sarcopenia vary across definition based on a single criterion (e.g. lean mass), but that variance was also apparent within the same definition if different accepted criteria were used (e.g. ‘standard’ ALM versus when adjusted for BMI). Of note, we were surprised that up to 17% of YF and 11% of YM were classed as sarcopenic using lean mass alone.

The directionality of difference between definitions is also not consistent, adding further to the challenge of translating sarcopenia research to inform on clinical practice. For example, wide variability in the prevalence of sarcopenia when using measures of muscle mass was identified in a study of 4000 community-dwelling older Chinese men and women, with the IWGS definition identifying the highest number of participants as sarcopenic [11]. In contrast, other work has reported that the EWGSOP definition identified the greatest number of individuals as sarcopenic [9, 12]; a finding that is echoed by the data reported herein.

Prevalence of sarcopenia based only on lean mass changed markedly when corrected for other physiological parameters (i.e. height or BMI). There is ongoing debate surrounding whether ALM is best adjusted using height, weight or BMI [10], although it is important to recognise that these corrections are based on limited data. For example, the criteria for ASMI adjusted by height used by the EWGSOP2 are based on *t*-scores from a single study of ~ 1500 participants aged 10–93 [14]. By comparison, other criteria within this definition are much more robustly evidenced, with the criteria for HGS drawn from 12 different studies of nearly 50,000 participants [15]. Given the clear rationale of correcting ALM measurements for physiological variance, more data are required to ensure these approaches are adequately evidenced and robust.

Overall, the classification of sarcopenia as a disease has increased demand for researchers and clinicians to develop approaches to prevent and treat sarcopenia. Although this is underway, claims on efficacy and effectiveness may be challenged by a lack of clarity on sarcopenia definitions and the contributing criteria, as highlighted in this paper. A single definition with well-defined easy-to-assess criteria would provide confidence in reported sarcopenia prevalence data and aid in research-led practice to hopefully improve the health-span of an ageing population.

## Data Availability

The datasets generated during the current study are available from the corresponding author on reasonable request.
